# Comprehensive Profiling Reveals Prognostic and Immunogenic Characteristics of Necroptosis in Soft Tissue Sarcomas

**DOI:** 10.3389/fimmu.2022.877815

**Published:** 2022-05-18

**Authors:** Lin Qi, Ruiling Xu, Xiaolei Ren, Wenchao Zhang, Zhimin Yang, Chao Tu, Zhihong Li

**Affiliations:** ^1^Department of Orthopedics, The Second Xiangya Hospital, Central South University, Changsha, China; ^2^Hunan Key Laboratory of Tumor Models and Individualized Medicine, The Second Xiangya Hospital, Changsha, China; ^3^Department of Microbiology, Immunology & Molecular Genetics, UT Health Science Center, University of Texas Long School of Medicine, San Antonio, TX, United States

**Keywords:** necroptosis, soft tissue sarcomas, immune, gene signature, risk score

## Abstract

Soft tissue sarcomas (STSs) are heterogeneous malignancies derived from mesenchymal cells. Due to its rarity, heterogeneity, and limited overall response to chemotherapy, STSs represent a therapeutic challenge. Necroptosis is a novel therapeutic strategy for enhancing immunotherapy of cancer. Nevertheless, no research has explored the relationship between necroptosis-related genes (NRGs) and STSs. In this study, differentially expressed NRGs were identified using The Cancer Genome Atlas (TCGA) and The Cancer Genotype-Tissue Expression (GTEx) project. The expression levels of 34 NRGs were significantly different. Several key NRGs were validated using RT-qPCR and our own sequencing data. Patients with STSs were divided into two clusters using consensus cluster analysis, and significant differences were observed in their survival (*p*=0.002). We found the differentially expressed genes (DEGs) between the two clusters and carried out subsequent analysis. The necroptosis-related gene signatures with 10 key DEGs were identified with a risk score constructed. The prognosis of TCGA-SARC cohort with low necroptosis-related risk score was better (p<0.001). Meanwhile, the low-risk group had a significantly increased immune infiltration. Using the data of GSE17118 and another immunotherapy cohort as external validations, we observed significant survival differences between the two risk groups (*p*=0.019). The necroptosis-related risk score proved to be an independent prognostic factor, and a nomogram was further established and integrated with other clinical features. Notably, the necroptosis-related gene signature could also act as the prognostic indicator in other malignancies based on pan-cancer analysis. In summary, the study outlines NRGs in STSs and their potential role in prognosis and will be one of the important directions for future research.

## Introduction

Soft tissue sarcomas (STSs) are heterogeneous malignancies derived from mesenchymal cells, with extensive clinical behaviors and pathological characteristics (1, 2). Liposarcoma is one of the most common types (3–5). STSs are relatively rare, accounting for approximately 1% of all adult cancers (6). Due to its rarity, heterogeneity, and limited overall response to chemotherapy, STSs represent a therapeutic challenge (7). Surgical excision is the mainstay of current treatment options, sometimes accompanied by radiotherapy and chemotherapy (8). Highly diverse genomic changes and low response rates to conventional therapies make it necessary to develop effective therapies for STSs (5).

Necroptosis is one of the programmed cell death mechanisms that can protect the body from cancer (9), which also has emerged as a novel therapeutic strategy to enhance cancer immunotherapy (10). Necroptosis is mainly mediated by receptor-interacting serine/threonine-protein kinase (RIPK) 1, RIPK3, and mixed lineage kinase domain-like pseudokinase (MLKL) (9). RIPK1 plays a key role in necroptosis and can be inhibited by necrostatin-1 (Nec-1), which was defined for the first time as a specific inhibitor of necroptosis (11). Down-regulation of the expression of the key regulatory factors of necroptosis is common in tumors, which suggests the potential association with tumors escaping from necroptosis and surviving (9). In addition to the above factors, tumor necrosis factor receptor 1 (TNFR1), FAS (CD95), and death receptor 4/5 (DR4/5, TRAIL-R1/2) have been reported to be key factors in necroptosis (12). Some of these effectors can also be activated by pathogen-associated molecular pattern molecules (PAMPs) and DNA damage and other necroptotic triggers (13).

Tumor immune escape is a significant marker of tumor occurrence, so restoring anti-tumor immunity becomes a novel therapeutic method (14). Nevertheless, the function of necroptosis in tumors is still disputed (10). Some types of cancer cells, such as HeLa cells, escape from necroptosis and survive because the expression of key regulators of necroptosis is down-regulated (15–17). Necroptosis can cause a strong adaptive immune response that can resist tumor progression. Nevertheless, involvement of the inflammatory response may also promote tumor growth and metastasis (9).

At present, there have been a few signature models for the prognosis of STS, based on the ferroptosis-related, pyroptosis-related, and glycolysis-related genes (18–20). Tumor heterogeneity is extremely complex on many levels, especially in soft-tissue sarcomas, so it is necessary to explore tumors from various research directions. Besides, a certain number of studies had limitations including unsystematic modeling methods, the lack of further analysis with related biological processes, and external validation (18–20). This prompted us to thoroughly investigate and develop a more reliable prognostic model of STS.

Moreover, there was no research on the function of necroptosis in STSs. In this study combined with genomics and clinical data, we not only constructed a prognostic model based on necroptosis-related genes, but also focused on the association between the tumor microenvironment and STS and used several cohorts for external validation. In high- and low-risk groups identified based on rigorous methods, the response to immunotherapy is significantly different, which is helpful for the clinical screening of patients. The findings demonstrated that the potential role of necroptosis in shaping TME and immune characteristics, which had significant implications for therapeutic guidance.

## Materials and Methods

### Sources of Data and Collection

Gene expression profiles were downloaded in the University of California Santa Cruz (UCSC) Xena Browser (https://xenabrowser.net/datapages/), including The Cancer Genome Atlas-Sarcoma (TCGA-SARC) cohort and normal tissues coming from The Cancer Genome Atlas (GTEx) cohort [42]. The RNA-seq data are log_2_ transformed FPKM values [log_2_(x+1)]. Next, the data from both databases were harmonized and cleaned according to equally rigorous steps, including reordering, quantification of gene expression, and removal of batch effects [27]. We collected clinical information (TCGA-SARC cohort) from the cBioPortal (https://www.cbioportal.org/). In total, 259 STS patients were obtained after data cleaning, including leiomyosarcoma (LMS, n=104), dedifferentiated liposarcoma (DDLPS, n=59), undifferentiated pleomorphic sarcoma (UPS, n=49), and myxofibrosarcoma (MFS, n=25), and other types (n=22) of STSs. Due to the lack of normal tissue in TCGA, gene expression profiling of 911 normal human adipose and muscle tissues in GTEx were integrated with the gene expression profiling in the TCGA queue. In addition, the data set GSE17118 in GEO was used for external verification, including the RNA-Seq configuration files and clinical information.

We also introduced the immunotherapy treated cohort. The cohort of melanoma patients treated with a combination of anti-PD-1 and anti-CTLA-4 was used to evaluate the association between the risk score and prognosis after immunotherapy (21).

### Identification of DEGs Between Necroptosis-Related Clusters

Based on previous studies (9–13, 22–28), a total of 36 NRGs have been identified and are listed in **Table S1**. The “limma” R package (Version 3.48.3) was used for identifying differentially expressed NRGs between the tumor and the normal tissue. DEGs were identified with | log_2_ (fold change) | >2 and false discovery rate (FDR) < 0.01 set in the expression value. We used the “Corr” R package (Version 0.4.3) to set up the correlation network for the NRGs with a correlation coefficient of 0.4. Visualization of somatic mutations in NRGs was conducted by using the “Maftools” R package (Version 2.8.0). The position of NRGs on the chromosome was shown by the Circos diagram through the “Circos” R package (Version 1.2.1).

### Protein-Protein Interaction (PPI) Network for NRGs

We found protein networks and key genes through PPI network analysis. (https://string-db.org/) (29). Cytoscape software (version 3.8.2) was used to further analyze the network of NRGs. Next, we set indexes including degree cutoff = 2, node score cutoff = 0.2, K-core = 2 and maximum depth = 100, and screened the hub genes according to MCODE.

### Necroptosis-Based Consensus Clustering Analysis

The consensus cluster analysis was performed using the “ConsensusClusterPlus” R package (Version 1.56.0), and the necroptosis-related subtypes of STSs were classified. Since K-means clustering analysis is random, the number of repetitions was set to 5000 to guarantee the stability of classification (30). The inertia calculation of K-means is the sum of the mean square distance of each sample from the nearest cluster center. Generally, the smaller the inertia, the better the model, but with the increase of K value, the rate of inertia decline was very slow (31). Besides, the proportion of ambiguously clustered pairs (PAC) was introduced to determine optimal K value (32). Kaplan-Meier (KM) curve was performed to visualize the differences in survival between cluster 1 (C1) and cluster 2 (C2) by using “survival” (Version 3.2-11) with “survminer” (Version 0.4.9) R packages.

### Gene Set Enrichment Analysis

We searched for DEGs between C1 and C2 according to necroptosis-related consensus clustering. Two distinct clusters were compared to find the corresponding DEGs. Based on “clusterProfiler” R package (Version 4.0.4), we carried on the Gene Ontology (GO) and Kyoto Encyclopedia of Genes and Genomes (KEGG) enrichment analysis of all DEGs, followed by the enrichment analysis of up- and down-regulated genes, respectively.

### Prognostic Model Development

Univariate Cox regression was used to evaluate the effect of DEGs on prognosis in different clusters. DEGs with significant effect on survival rate were screened (*p* < 0.01), which were further entered into the least absolute shrinkage and selection operator (LASSO) Cox regression analysis to narrow down the scope of gene selection using “glmnet” R package (Version 4.1-2). The risk score was further built based on the formula:


$$\sum_{i}^{n}X_{i}*Y_{i}\left(X_{i}:coefficients\;of\;the\;gene\;i,\cdot Y_{i}:expression\;values\;of\;the\;gene\;i\right)$$

The TCGA-SARC cohort was classified into two groups according to the median risk scores. The survival of the high-risk and low-risk groups in the TCGA-SARC cohort was illustrated using KM plots. For further external validation, GSE17118 was brought into the analysis and these patients were divided into similar groups according to the same critical point. The “timeROC” R package (version 0.4) was used to evaluate the correctness of predictions. In addition, a multivariate Cox regression analysis was performed to analyze the risk score combined with clinical characteristics such as age, gender, metastasis, tumor site, multifocality, and tumor depth. The predictive model was illustrated using the nomogram and further evaluated with calibration curves.

A similar approach was used to compare the glycolysis-related signature model with the model in the present study. The glycolysis-related DEGs were obtained from Y. Liu et al. and were modeled based on TCGA-SARC cohort. GSE17118 was also used for external validation. The timeROC R package (version 0.4) was used to evaluate the correctness of predictions.

### ssGSEA and Immune Infiltration Analysis

Using the “GSVA” (Version 1.40.1) and “gsease” (Version 1.54.0) R packages, 16 immune cell infiltrations and 13 related functions of necroptosis-related risk groups in the TCGA-SARC cohort and GSE17118 were analyzed by ssGSEA quantitative analysis. The correlation between DEG expression and immunologic invasion was analyzed by the Tumor Immune Estimation Resource 2.0 database (TIMER2.0) (33).

The analysis of Tumor Immune Dysfunction and Exclusion (TIDE) was used to predict the response of STS patients to immunotherapy (34).

### Chemotherapeutic Response Prediction

The chemotherapeutic response for each group was predicted according to the largest publicly available pharmacogenomics database [the Genomics of Drug Sensitivity in Cancer (GDSC), https://www.cancerrxgene.org/]. We screened eight commonly used chemotherapy drugs including Doxorubicin, Cytarabine, Docetaxel, Methotrexate, Sunitinib, Gefitinib, Lapatinib, and Bortezomib. The prediction process adopted “*pRRophetic*” R package, and ridge regression is used to estimate half of the maximum inhibitory concentration (IC50). The prediction accuracy was evaluated by ten-fold cross-validation based on the GDSC training set. All parameters were default values, excluding the batch effect of “combat” and “allSoldTumours,” and repeat gene expression was replaced by average values (35, 36).

### Cell Culture and Cell Lines

The human skin fibroblast cell line (HSF) along with their culture media were obtained from Fenghui Biotechnology Co., Ltd (Hunan, China). The human synovial sarcoma cell line (SW-982) was provided by the American Type Culture Collection (ATCC). The human liposarcoma cell line (SW-872) was purchased from Procell Life Science&Technology Co., Ltd. The primary human synovial sarcoma cells (hSS-005R) were established for the validation of NRGs. SW-982, SW-872, and hSS-005R were cultured in Dulbecco’s modified Eagle medium (DMEM) (Gibco, United States), which was supplemented with 1% penicillin-streptomycin (NCM Biotech, China) and 10% fetal bovine serum (FBS) (Gibco, United States). Cells were cultured in a humidified incubator (Thermo Fisher Scientific, United States) at 37°C and 5% CO_2_.

### Full-Length Transcriptome Analysis

Full-length transcriptome analysis was performed by Biomarker Technologies (Biomarker Technologies Ltd, Beijing, China). All operations were in accordance with Oxford Nanopore Technologies (Oxford Nanopore Technologies, Oxford, United Kingdom). The analysis platform (BMKCloud) performs correlation analysis based on reference sequences and nanopore transcriptome sequencing data.

### Quantitative Real-Time PCR

Total cellular RNA was isolated with RNA Express Total RNA Kit (M050, NCM Biotech, China) (37). The RNA was reverse transcribed using the RevertAid First Strand cDNA Synthesis Kit (Thermo). We performed RT-qPCR on the StepOne Plus (Applied Biosystems, United States) by utilizing SYBR Green qPCR Master Mix (2×) (Bimake, United States). The primers are listed in **Table 1**.

**Table 1:** Sequences of the primers used in RT-qPCR.

Gene	Sequence of primer
*CHMP4B*	F: AGAAGCACGGCACCAAAAAC
	R: GCTGGAACTCGATGGTTGATAAT
*HMGB1*	F: TATGGCAAAAGCGGACAAGG
	R: CTTCGCAACATCACCAATGGA
*TNFAIP3*	F: TCCTCAGGCTTTGTATTTGAGC
	R: TGTGTATCGGTGCATGGTTTTA
*GADPH*	F: CAGGAGGCATTGCTGATGAT
	R: GAAGGCTGGGGCTCATTT

### Statistical Analysis

We used R (version 4.0.1) for data analysis. Wilcoxon rank-sum test was performed to determine differential gene expression between the two groups, and the *p* value of each gene was calculated. For survival analysis, the log-rank test and KM curve were performed. For correlation analyses of gene expression, Spearman’s correlation test was performed. To evaluate clinical characteristics between two groups, χ2 test (or Fisher’s exact test) was performed. Multivariable analysis was performed with Cox regression analysis to evaluate prognostic factors and calculate the hazard ratio (HR) with a 95% confidence interval (CI). A statistical difference was considered to be significant as *p<0.05, **p<0.01 and ***p<0.001.

## Results

### Identification of NRGs Between STSs and Normal Tissues

**Figure 1:**
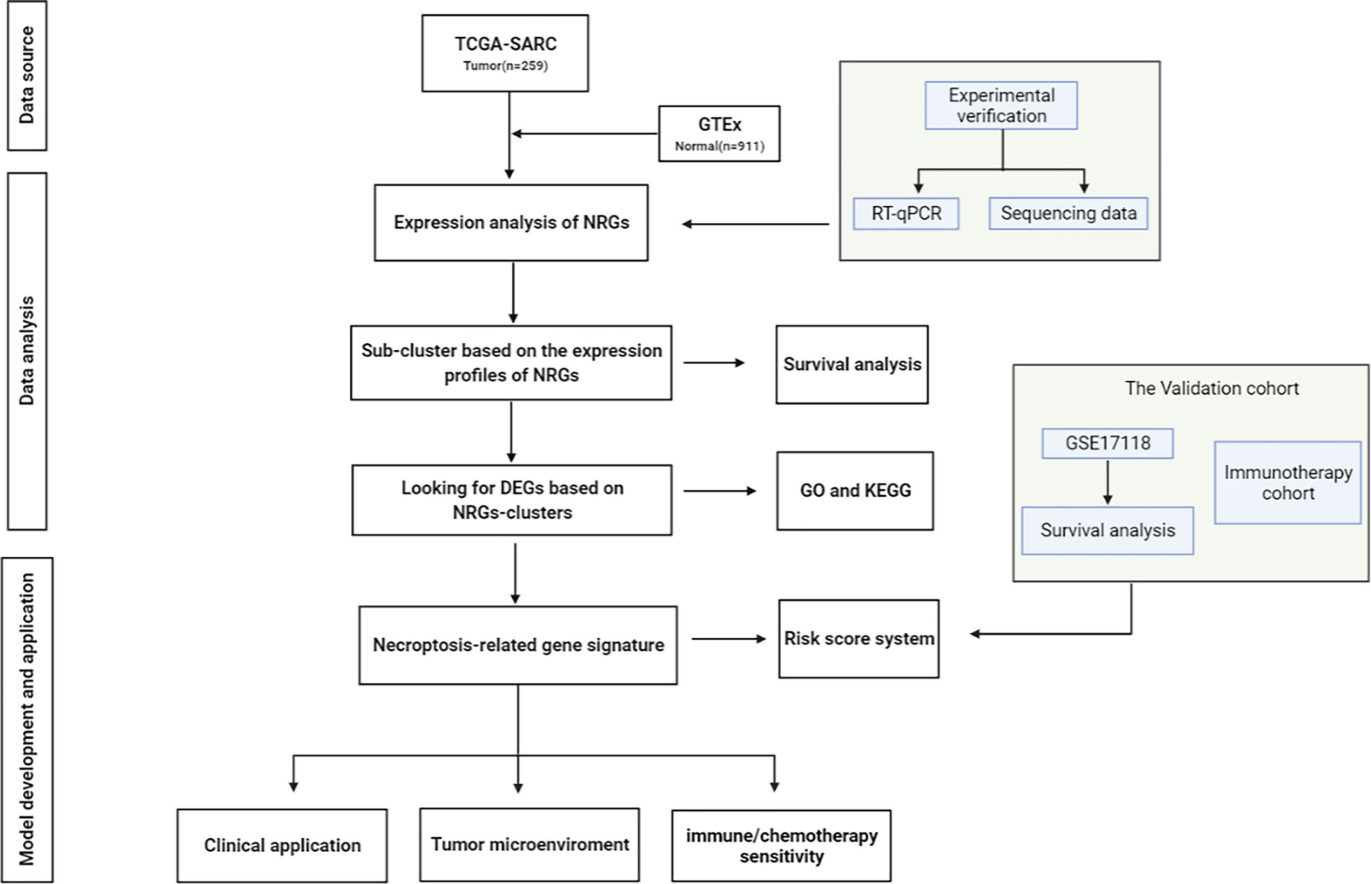
Study design. Overall idea and flow chart.

**Figure 2:**
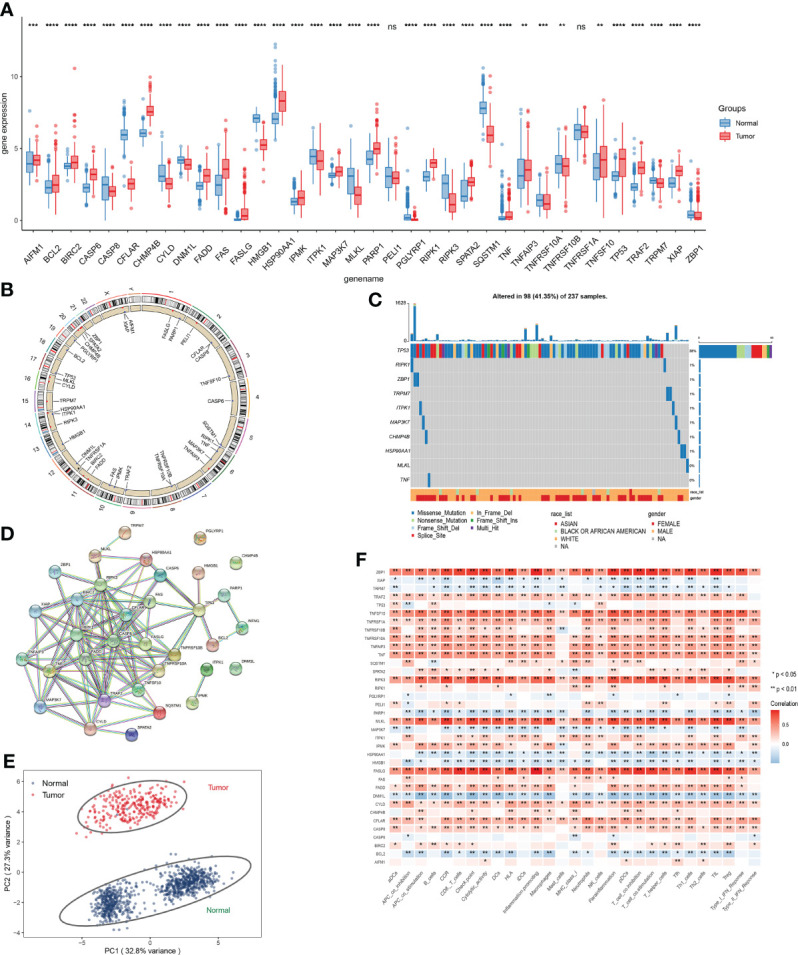
Expression analysis of NRGs. **(A)** Expression level of NRGs between STSs and normal tissue. **(B)** The location of NRGs on chromosomes. **(C)** Frequency and type of mutations in NRGs. **(D)** Based on NRGs encoded proteins, the PPI network was constructed according to the interaction score of 0.9. **(E)** Principal component analysis (PCA) based on NRGs to distinguish STSs from normal tissues. **(F)** The correlation of the expression level of NRGs and immune cells. ns, p ≥ 0.05; *, 0.01 ≤ p < 0.05; **, 0.001 ≤p < 0.01; ***, 0.0001 ≤p < 0.001; ****p < 0.0001.

**Figure 3:**
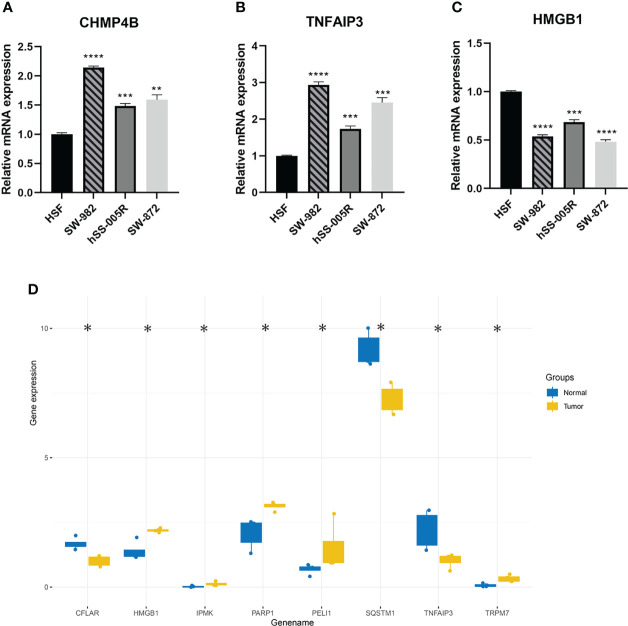
**(A–C)** Validation of CHMP4B, TNFAIP3, HMGB1 expression in cell lines by using RT-qPCR. **(D)** The differential expression of NRGs was verified by sequencing data. ns, p ≥ 0.05; *, 0.01 ≤ p < 0.05; **, 0.001 ≤p < 0.01; ***, 0.0001 ≤p < 0.001; ****p < 0.0001.

The visual flowsheet of the study is present in **Figure 1**. All 36 NRGs were analyzed between STSs and normal tissues from two databases (TCGA-SARC and GTEx, **Table S2**). There were significant differences in the expression of 34 of the 36 NRGs (*p* < 0.05). There were 21 genes (AIFM1, BCL2, BIRC2, CASP6, CHMP4B, FADD, FAS, FASLG, HSP90AA1, IPMK, MAP3K7, PARP1, RIPK1, SPATA2, TNF, TNFAIP3, TNFRSF1A, TNFSF10, TP53, TRAF2 and XIAP) upregulated and 13 genes (CASP8, CFLAR, CYLD, DNM1L, HMGB1, ITPK1, MLKL, PGLYRP1, RIPK3, SQSTM1, TNFRSF10A, TRPM7, and ZBP1) downregulated in STS tumor samples (**Figure 2A** and **Figure S1A**). The NRGs expression within HSF, SW-982, hSS-005R, and SW-872 cell lines were quantified by RT-qPCR in order to verify the expression level of some key NRGs. As shown in **Figures 3A–C**, compared with human skin fibroblast cell line (HSF), CHMP4B, TNFAIP3 levels were statistically significantly higher and HMGB1 was lower in synovial sarcoma and liposarcoma cell lines. Besides, we validated NRGs expression levels with our own sequencing data (four tumor patients, four normal controls, **Table S3**). As shown in **Figure 3D**, CFLAR, SQSTM1, and TNFAIP3 were downregulated in tumors, and HMGB1, IPMK, PARP1, PELI1, and TRPM7 were upregulated in tumors. Furthermore, the expression levels of CFLAR, PARP1, IPMK, and SQSTM1 in patients were consistent with the results obtained from the public database.

The correlation network of 34 NRGs was demonstrated in **Figures S1B, C**. The location of 34 NRGs on chromosomes was shown in **Figure 2B**. Moreover, genetic alterations of NRGs were analyzed (**Figure 2C**). Ninety-eight of 237 (41.35%) sarcoma samples showed necroptosis-related mutations, and TP53 was the most frequently mutated gene. Through the establishment of PPI network, 10 hub genes (RIPK1, CASP8, RIPK3, TRAF2, FADD, BIRC2, CFLAR, TNFRSF10A, TNF, and TNFRSF10B) were identified (**Figure 2D**). In addition, these 34 NRGs can effectively distinguish STSs from normal tissues at the expression level (**Figure 2E**). We detected the correlations among the expression level of NRGs and immune-related cells, which uncovered that ZBP1, TNFSF10, RIPK3, MLKL, FASLG, and CYLD significantly correlated with immune-related cells (*p* < 0.05) (**Figure 2F**).

### Identification of TCGA-SARC Clustering Based on Necroptosis

**Figure 4:**
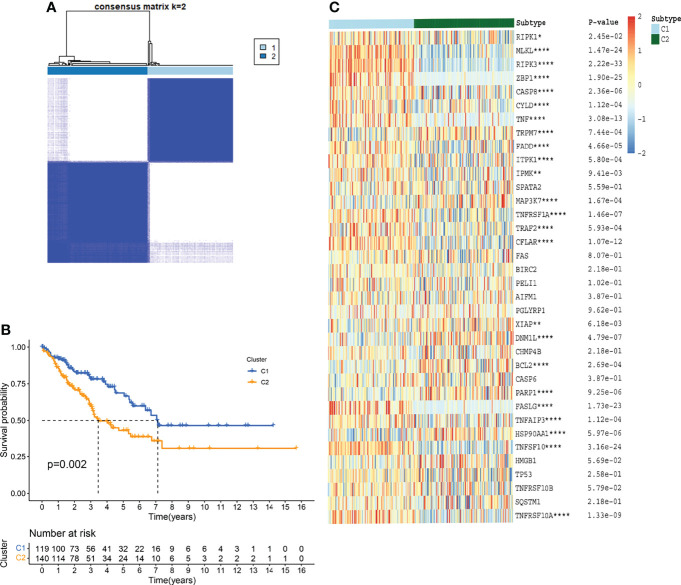
TCGA-SARC clusters based on NRGs through consensus clustering analysis. **(A)** TCGA-SARC cohort was divided into two distinct clusters (k=2, repetition=5000) **(B)** Overall survival (OS) curve of two clusters **(C)** Heatmap of NRGs among two clusters. ns, p ≥ 0.05; *, 0.01 ≤ p < 0.05; **, 0.001 ≤p < 0.01; ***, 0.0001 ≤p < 0.001; ****p < 0.0001.

For evaluating the clinical features and prognosis of different STSs subtypes based on necroptosis regulators, consensus clustering analysis was performed to assign the TCGA-SARC cohort into distinct clusters (**Figure 4A** and **Figures S2A–F**). The optimal K was determined as the PAC analysis detected across most of the tested pairs. The TCGA-SARC cohorts were divided into two distinct clusters. The C1 contains 119 STS patients and C2 contains 140 STS patients. Remarkably, the overall survival curve was significantly different between C1 and C2 (*p*=0.002, **Figure 4B**). In **Figure 4C**, the NRG expression levels in two different clusters were demonstrated.

### Profiling DEGs Between Two Distinct Clusters

**Figure 5:**
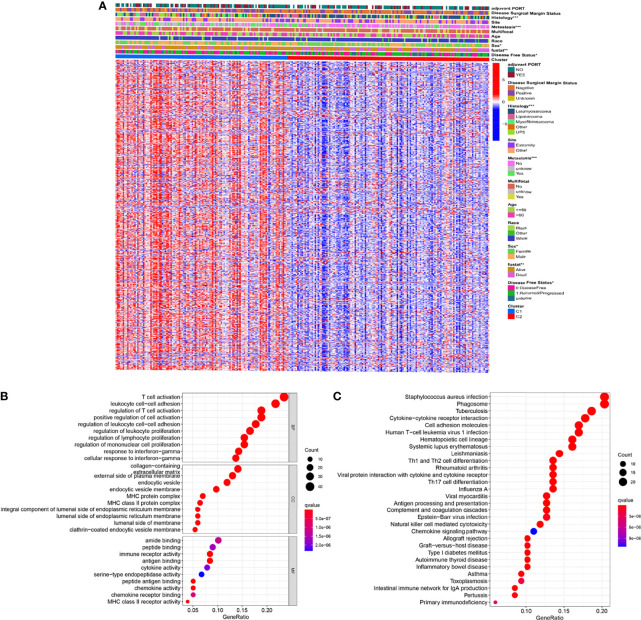
Identification of DEGs between the two clusters **(A)** Heatmap showing DEGs and corresponding clinical features **(B)** GO enrichment analysis including biological process (BP), cellular component (CC), and molecular function (MF). **(C)** KEGG enrichment analysis indicating related genes and pathways. ns, p ≥ 0.05; *, 0.01 ≤ p < 0.05; **, 0.001 ≤p < 0.01; ***, 0.0001 ≤ p < 0.001; ****p < 0.0001.

Take | log_2(_fold change) | > 2 and FDR < 0.01 as the stringent selecting criterion, there were a total of 565 DEGs in C1 versus C2 (**Figure 5A** and **Table S5**). We found significant differences in clinicopathological features including histology, metastasis, sex, vital status, and disease-free status between two distinct clusters (*p* < 0.05).

Subsequently, the GO and KEGG enrichment analyses were performed on these 565 DEGs to reveal the pathways and possible mechanisms of necroptosis-related clusters. Through GO enrichment analysis we could find that DEGs were concentrated in T cell activation, leukocyte cell-cell adhesion, and regulation of T cell activation (**Figure 5B**). Moreover, KEGG indicated that DEGs were also significantly enriched in staphylococcus aureus infection, phagosome, and tuberculosis (**Figure 5C**). Further, GO and KEGG enrichment analyses of up-regulated and down-regulated DEGs were performed, respectively (**Figures S3A–D**).

### Establishment of Necroptosis-Related Gene Signature

**Figure 6:**
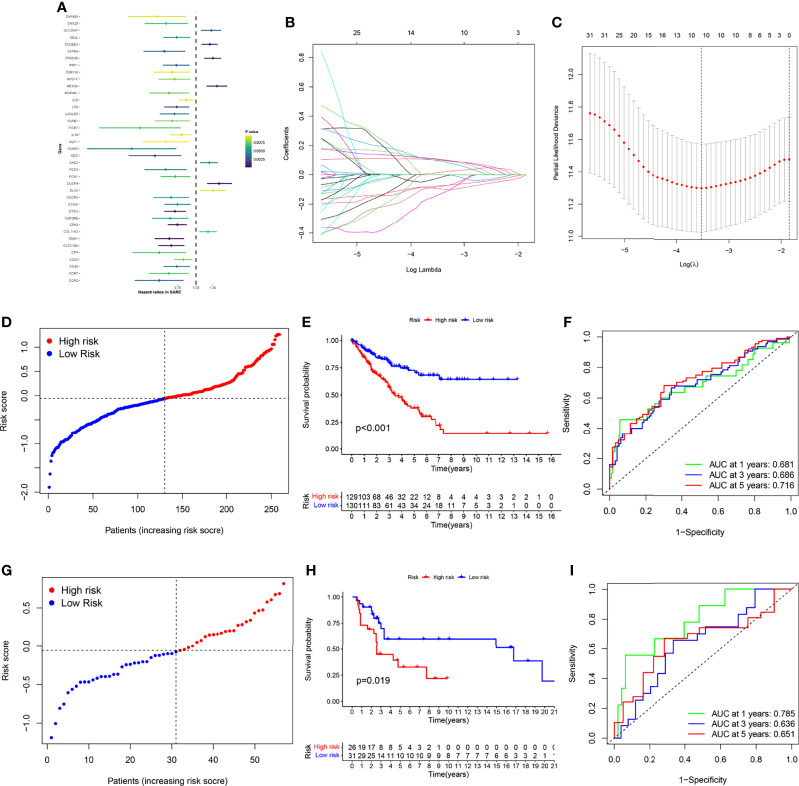
Gene signature’s development and validation. **(A)** 39 DEGs with significant prognostic differences using univariate Cox regression analysis. **(B)** LASSO regression analysis of 39 DEGs **(C)** Cross-validation method to select optimal genes. **(D)** TCGA-SARC cohort was distributed by risk score. **(E)** OS curve of TCGA-SARC cohort. **(F)** Evaluate the prognostic performance of the risk score using time-dependent ROC in TCGA-cohort. **(G)** GSE17118 cohort was distributed by the risk score. **(H)** Disease-free survival (DFS) curve of GSE17118 cohort. **(I)** Evaluate the prognostic performance of the risk score using time-dependent ROC in the GSE17118 cohort.

Univariate Cox regression was used to analyze the effect of 565 DEGs on prognosis. Taking p value less than 0.01 as the significant difference, 39 genes were strictly screened for further analysis (**Figure 6A**). Finally, using Lasso Cox regression analysis, 10 key genes were identified from 565 DEGs, and gene signatures related to necroptosis were constructed (**Figures 6B, C**). The formula for calculating the risk score was (-0.08461 * CFP exp.) + (-0.09298 * CTSG exp.) + (0.09801 * DUSP9 exp.) + (-0.14346 * ITGB7 exp.) + (0.01339 * MEX3A exp.) + (-0.07727 * CPA3 exp.) + (0.10592 * SLC26A7 exp.) + (0.10824 * SCUBE3 exp.) + (-0.02635 * CMA1 exp.) + (0.10008 * PRSS35 exp.). We then divided 259 patients with STSs into a low-risk group with 130 patients and a high-risk group with 129 patients, as the median was selected as the split point (**Figure 6D** and **Figure S4A**). Principal component analysis (PCA) and t-distributed stochastic neighbor embedding (t-SNE) can clearly distinguish the two groups (**Figures S4C, E**). In the TCGA-SARC cohort, the KM plot showed that there was a significant difference between the two risk scoring groups (*p*<0.001, **Figure 6E**). The area under the curve (AUC) of the time-dependent receive operating characteristic (ROC) curve was used to evaluate the performance of the model (one-year [AUC]=0.681, three-year [AUC]=0.686, and five-year [AUC]= 0.716, **Figure 6F**).

For verifying the accuracy of the necroptosis-related gene signature, we calculated the risk score of GSE17118 obtained from the GEO database. The same segmentation method for the TCGA cohort was used in GSE17118, which was also separated into two risk score groups (**Figures 6G** and **S4B**). PCA and t-SNE also illustrated the optimal degree of discrimination between the two groups of GSE17118 (**Figure S4D, F**). We also performed survival analysis and AUC calculation. It is worth noting that there was a significant difference in disease-free survival (DFS) between the two different risk groups (*p* = 0.019, **Figure 6H**), and the AUC was relatively satisfactory (one-year [AUC]= 0.785, three-year [AUC]= 0.636, five-year [AUC]= 0.651, **Figure 6I**). Then we performed KM survival analysis for different subgroups (**Figures S5A–K**). Subgroup analysis of the risk scores in different clinical characteristics groups in the TCGA-SARC cohort also yielded stable results. In addition, we built a glycolysis-related prognostic model for comparison based on Y. Liu’s research (19) (**Figures S6A–D**). Although the Glycolysis-related signature could identify patients with poor prognosis in the TCGA-SARC cohort, this signature did not seem to be ideal for the external validation with low AUC. Besides, we were surprised to find thaõt the necroptosis-related gene signature could also be applied to predict the prognosis of other cancers including liver cancer (LIHC), lung adenocarcinoma (LUAD), kidney clear cell carcinoma (KIRC), head and neck cancer (HNSC), colon cancer (COAD), endometrioid cancer (UCEC), and kidney papillary cell carcinoma (KIRP) in the TCGA database (**Figures S8A–G**).

### Establishment of Necroptosis-Related Prognostic Model

**Figure 7:**
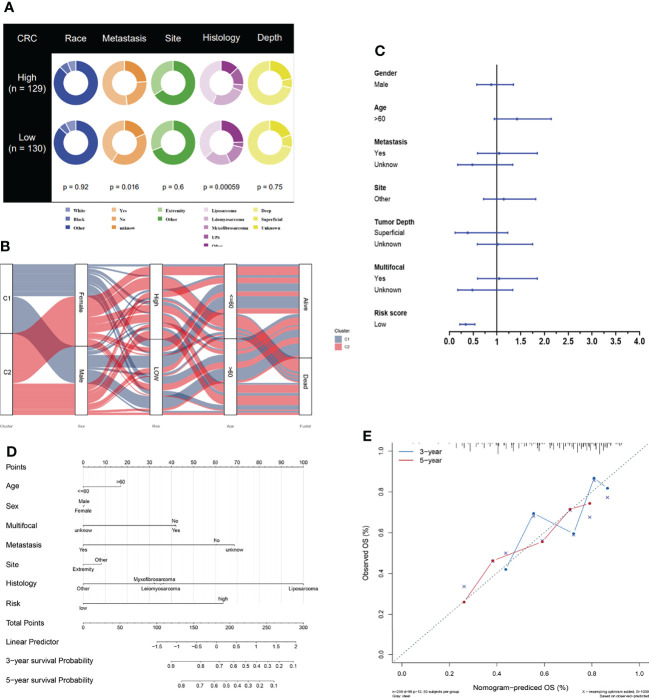
Development of necroptosis-based prognostic model. **(A)** Clinical characteristic between low-risk and high-risk groups. **(B)** Alluvial diagram illustrating the relationship of necroptosis-based cluster distribution, clinical characteristics, different risk groups and survival outcomes. **(C)** Multivariate Cox regression analysis of clinical characteristics and necroptosis-based risk score. **(D)** Nomogram predicting 3-years and 5-years survival rate of STS patients. **(E)** Calibration curve for predicting OS rate of STS patients.

To further study the potential value of its clinical application, we analyzed the risk score combined with other clinical characteristics. The clinical features of race, metastasis, histology site, and tumor depth between the two risk groups are demonstrated in **Figure 7A**. The alluvial diagram also illustrates the relationships among the cluster distribution, clinical features, risk score, and survival based on necroptosis regulators (**Figure 7B**). In addition, a multivariate Cox regression analysis was performed, combining clinical features with a necroptosis-related risk score, to establish a prognostic model (**Figure 7C** and **Table S4**). Based on the necroptosis-related model, we further created the novel nomogram that had clinical utility and was complementary to the necroptosis-related model (**Figure 7D**). The calibration curve of the nomogram has proven that the 3-year and 5-year OS rates are relatively well predicted (**Figure 7E**).

### Association Between Immune Infiltration and Risk Score

**Figure 8:**
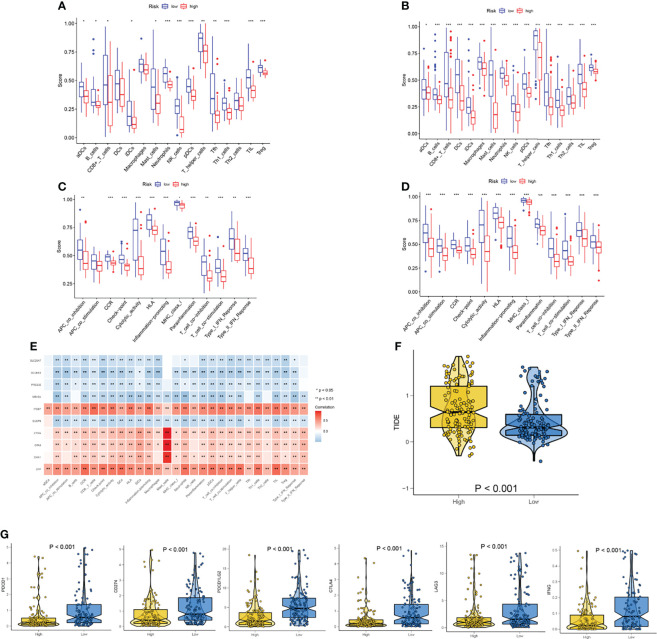
Analysis of immune status based on necroptosis-related risk score. **(A, C)** Comparisons of immune cells and immune functions between different risk groups in TCGA-SARC cohort. **(B, D)** Comparisons of immune cells and immune functions between different risk groups in GSE17118 cohort. **(E)** The correlation of the expression level of signature genes and immune cells. **(F)** The differences in the TIDE score between two risk groups. **(G)** The differences in the expression of checkpoint and related immune genes between two risk groups. ns, p ≥ 0.05; *, 0.01 ≤ p < 0.05; **, 0.001 ≤p < 0.01; ***, 0.0001 ≤p < 0.001; ****p < 0.0001.

To compare immune activity between high- and low-risk groups, we analyzed immune infiltration and function of the population by ssGSEA. The degree of infiltration of immune cells and pathways in the cohort could be assessed according to the ssGSEA. In the TCGA-SARC cohort, the immune cell infiltration in the high-risk group was significantly lower than that in the low-risk group (*p*<0.05, **Figure 8A**). All 13 related immune functions were also significantly decreased in the high necroptosis-related risk group (*p*<0.05, **Figure 8B**). The cohort of GSE17118 was also analyzed for immune activity in the same approach. Similar to the immune status of most tumors, the immune infiltration of the high-risk group decreased significantly (**Figures 8C, D**). The coherence of key DEGs in the gene signature and immune infiltration was also illustrated (**Figures S7A–E**). We also detected the correlations between the expression level of signature genes and immune-related cells. Among them, ITGB7 and CFP showed a significant positive correlation with all immune-related cells (*p*<0.05) (**Figure 8E**). The expression of the checkpoint and related immune genes between the two risk groups was also compared (**Figure 8G**). The groups with lower risk scores had significantly higher levels of related gene expression than the high-risk group.

**Figure 9:**
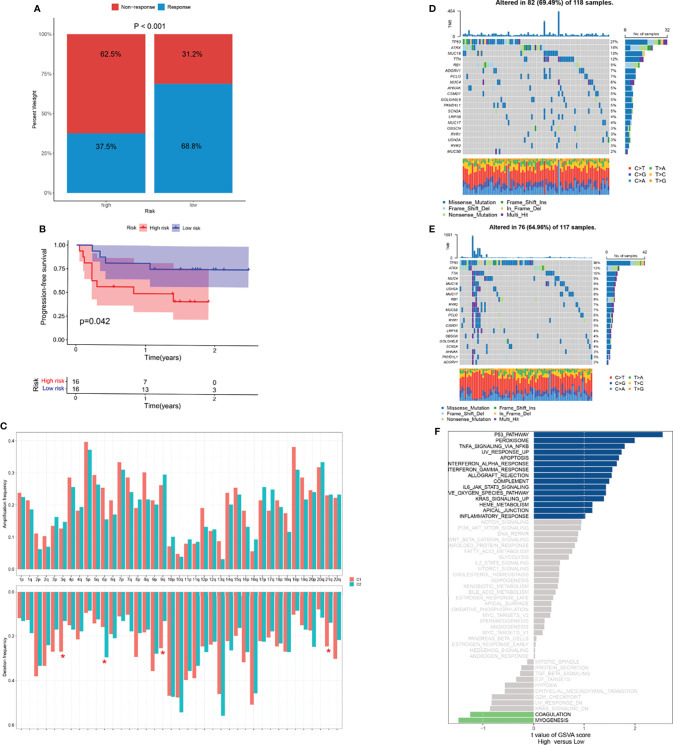
Potential role of risk score in mutation and immunotherapy. **(A)** The proportion of clinical response to anti-PD-1 with anti-CTLA-4 immunotherapy in two risk groups in the melanoma cohort. **(B)** The progression-free survival (PFS) curve comparing survival of two risk groups in a cohort of melanoma patients treated with combination of anti-PD-1 and anti-CTLA-4. **(C)** The frequency of arm-level amplification and deletion between two risk groups. **(D, E)** The somatic mutation frequency of two risk groups in TCGA-SARC cohort. **(F)** Differences in pathway activities between two risk groups. *p < 0.05; ns, p ≥ 0.05.

The significant correlation between risk score and immune infiltration prompted us to further explore the role of the risk score in immunotherapy. The TIDE analysis revealed that the high-risk group had significantly higher TIDE scores (**Figure 8F**). Because there was no immunotherapy information in the TCGA-SARC cohort, a cohort of melanoma patients treated with a combination of anti-PD-1 and anti-CTLA-4 was introduced. The risk scores of patients in this cohort were calculated based on the above analysis and the same cut-off point. We surprisingly found that the response rates to immunotherapy were significantly higher in the lower-risk group (**Figure 9A**). Moreover, patients in the lower-risk group had significantly better prognoses (**Figure 9B**).

### Potential Role of Risk Score in Biological Processes and Chemotherapeutic Value

For the frequency of somatic mutation, no significant differences were found between the two risk groups (**Figures 9D–E**). Of note, several arm-level copy number alterations were also exhibited between these two groups (**Figure 9C**). Pathways showing notable differential expression including *p53*, peroxisome pathways (**Figure 9F**).

**Figure 10:**
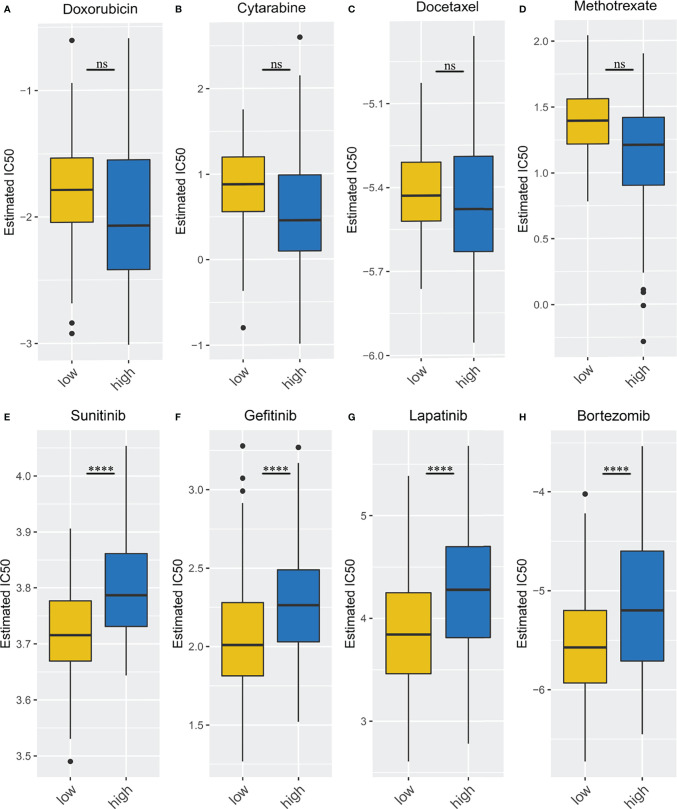
Different immune/chemotherapy sensitivity analysis. **(A–D)** the box plots of the estimated IC50 for Doxorubicin, Cytarabine, Docetaxel, Methotrexate; **(E–H)** the box plots of the estimated IC50 for Sunitinib, Gefitinib, Lapatinib, and Bortezomib. ****p < 0.0001; ns, p ≥ 0.05.

Because immune/chemotherapy is one of the common methods for the treatment of STSs, we tried to evaluate the response of two necroptosis-related subtypes to eight chemo-drugs including Doxorubicin, Cytarabine, Docetaxel, Methotrexate, Sunitinib, Gefitinib, Lapatinib, and Bortezomib. Therefore, we trained the predictive model on the GDSC cell line dataset by ridge regression with a satisfied predictive accuracy evaluated by 10-fold cross-validation. We estimated the IC50 for each sample in the TCGA-SARC cohort based on the predictive model of these eight chemo-drugs. We could observe significant differences in IC50 estimates in Sunitinib, Gefitinib, Lapatinib, and Bortezomib between the high- and low-risk groups, which suggested that the low-risk group was more sensitive (*p* < 0.001 for Sunitinib, Gefitinib, Lapatinib, and Bortezomib) (**Figures 10E–H**). However, compared with the high-risk group, none of the common chemotherapeutic drugs, including Doxorubicin, Cytarabine, Docetaxel, and Methotrexate, showed a significantly different response in the low-risk group (**Figures 10A–D**).

## Discussion

Necroptosis is widely regarded as an inflammatory form of cell death, which is related to the production of pro-inflammatory cytokines, the disruption of biofilm, and the release of intracellular damage-related molecular patterns (38). Therefore, the separation of programmed cell death depends on the molecular participants and the processes involved, and their functional significance in physiology and disease has begun to be clarified (39). Although necroptosis has been widely studied, there are few studies on the relationship between STS and necroptosis. In the current study, combined with the study of NRGs reported in studies of other tumors, 36 genes were consistent in necroptosis (22, 24).

Due to the limited number of normal tissues in the TCGA database, we combined TCGA and GTEx to compare the gene expression levels between tumors (TCGA database) and normal tissues (GTEx database) (40). Within differential expression-based NRGs sets, 34 NRGs offer major advantages conceived to distinguish normal tissues from tumors.

Consensus clustering is a commonly used method for the identification of tumor subtypes and survival patterns of malignant tumors (41, 42). According to the well-established theory, the optimal K was determined as 2 (32). Because the total sample size of TCGA-SARC was relatively limited (n=259) due to the nature of the database, clustering TCGA-SARC into more than three groups may lead to more limited sample sizes in each group. Thus, it is more difficult to screen a sufficient number of differentially expressed genes through multiple comparisons among many groups. This study subtyped TCGA-SACR and provided new insight into identifying biological differences in STSs according to NRGs. The prognosis of STS patients in necroptosis-related cluster 2 was significantly poorer, and most NRGs decreased significantly, including ZBP1, MLKL, and RIPK3. Meanwhile, these genes were significantly associated with infiltration of all immune cells, and low expression of the NRGs resulted in low infiltration of immune cells, which may lead to tumor immune escape (43). This was consistent with previous research, and ZBP1 was finally identified as a central mediator of RIPK3-MLKL mediated necroptosis through its direct interaction with RIPK3, which resulted in RIPK3 autophosphorylation and MLKL dependent necroptosis (44). Phosphorylation of RIPK1 and RIPK3 usually triggered signal transduction events that eventually led to necroptosis, but RIPK3 can drive necroptosis independent of RIPK1 activity (45). Therefore, the concentration of RIPK3 did not decrease in our results, either because of sample effects or because RIPK1 did not contribute much.

We further analyzed the DEGs between the two necroptosis-related clusters to develop a risk scoring system, which was also an effective method to identify the different risk levels of STS patients. Several immune-related pathways related to necroptosis were revealed by gene enrichment analysis, which was also consistent with its role in mediating the immune system (45). Importantly, necroptosis-related signatures were established based on 10 key DEGs. Significant survival differences in the TCGA-SARC cohort confirmed the utility of the necroptosis-related risk score. Surprisingly, the necroptosis-related risk score was also effective in predicting disease-free survival (DFS) for externally validated in the GSE17118 cohort. Besides, our model has better results in external validation than the glycolysis-related prognostic model.

In addition, we performed TIDE (34) analysis and introduced a cohort of melanoma treated with immunotherapy (21). It is noted that the high-risk group had significantly higher TIDE scores, which suggests potential therapeutic values in these patients. Numerous studies suggest that acquired or intrinsic resistance to necroptotic stimuli is considered a major hindrance to therapeutic success in malignant melanoma (46, 47). Therefore, the signature we established can be potentially utilized to predict the response to immunotherapy. To further explore the characteristics between high- and low-risk groups, we performed the mutation analysis in both groups. TP53 mutation has been associated with STSs in multiple reports (48–50). The TP53 pathway was also significantly different between subgroups, which further confirmed the application of our model.

Multivariate Cox analysis identified the necroptosis-related risk score as an independent prognostic factor in the OS of the TCGA-SARC cohort. In addition, we also developed the nomogram for clinical application combined with a necroptosis-related risk score and clinical indicators. The OS rate of STS patients can be estimated by summing the scores of each indicator. In the high-risk group, the degree of immune infiltration decreased significantly, which also showed abnormal immune functions (51).

Signal Peptide, CUB Domain And EGF Like Domain Containing 3 (SCUBE3), which is a gene associated with necroptosis signature, is a secretory cell surface glycoprotein, overexpressed in many tumors (52). Matrix metalloproteinase-2 (MMP-2) and MMP-9 cleavage by SCUBE3 releases fragments, which can bind to transforming growth factor-b (TGF-b) type II receptors, thereby activating the TGF-b signal transduction and promoting tumor development (53). SCUBE3 in osteosarcoma cell line U2OS and invasive lung cancer was overexpressed, which were inextricably linked to patient survival prognosis (52, 54). Another study was performed in HER2-positive breast cancer, where it was discovered that SCUBE3 also demonstrated a tendency to be overexpressed (53). In this research, the coefficient of SCUBE3 was positive in the formula of necroptosis-related risk score. Our results show that SCUBE3 is associated with low immunologic invasion. Overexpression of SCUBE3 may weaken the killing effect of the immune system on tumors and eventually lead to tumor metastasis, which plays the greatest role in the increase of risk score and may provide some directions for related fields.

The study selected four immunotherapy drugs (Sunitinib, Gefitinib, Lapatinib, and Bortezomib) and four conventional chemotherapy drugs (Doxorubicin, Cytarabine, Docetaxel, and Methotrexate) to test the response of different groups to chemotherapy and immunotherapy (55–62). It indicated that the high-risk group had a worse response to immunotherapy but not conventional chemotherapy, which may suggest that our grouping is more relevant to immunity, but it could also provide useful information on the effectiveness of immunotherapy.

Furthermore, we validated the results with our own sequencing data (four patients, four controls) and cell lines (HSF, SW-982, hSS-005, and SW-872). Surprisingly, both HMGB1 and TNFAIP3 showed significant differences in validation, but the results were reversed. The results of the cell experiments were consistent with the previous results, possibly due to the error caused by the small sample size of the sequencing data, which is worth further exploration.

To our knowledge, this was the first time to carefully explore the relationship between NRGs and STSs and conduct a multi-dimensional analysis from clinical features to immune infiltration. This study comprehensively and carefully analyzed the relationship between NRGs gene expression, clinical characteristics, and prognosis in STSs. There were 34 NRGs with significant expression differences, which can be used to effectively distinguish STS from normal tissues. Different clusters were divided and entered into further DEG analysis. Furthermore, a necroptosis-related risk score model with 10 core DEGs was extended and used as an independent prognostic factor in patients with STSs. Finally, we explored the relationship between immunity and grouping and found that there were also significant differences between immune infiltration.

## Data Availability Statement

The original contributions presented in the study are publicly available. This data can be found here: https://www.ncbi.nlm.nih.gov/geo/query/acc.cgi?acc=GSE198568.

## Author Contributions

ZL and CT contributed to the conception and made final approval of the version, LQ and RX performed study concept and design and wrote the manuscript. XR, WZ, and ZY helped with data analysis. All authors read and approved the final manuscript.

## Funding

This work was supported by grants from the National Natural Science Foundation of China (NSFC; No. 81902745, No.82172500, No.82103228), Hunan Provincial Research and Development Program in Key Areas (2019WK2071, 2020DK 2003), and China Postdoctoral Science Foundation (No. 2021M693557).

## Conflict of Interest

The authors declare that the research was conducted in the absence of any commercial or financial relationships that could be construed as a potential conflict of interest.

## Publisher’s Note

All claims expressed in this article are solely those of the authors and do not necessarily represent those of their affiliated organizations, or those of the publisher, the editors and the reviewers. Any product that may be evaluated in this article, or claim that may be made by its manufacturer, is not guaranteed or endorsed by the publisher.

## Supplementary Material

Supplementary Figure 1 Identification of NRGs between STSs and normal tissues. **(A)** NRGs-related heatmap of tumor tissue and normal tissue. **(B, C)** The correlation network of 34 NRGs.

Supplementary Figure 2 Consensus clustering analysis. **(A–D)** Consensus clustering based on regulator-related DEGs (K = 2-5). **(E)** The CDF Plot based on the DEGs. **(F)** Delta area plot of consensus clustering based on the DEGs.

Supplementary Figure 3 Gene set enrichment analysis. **(A)** GO enrichment analysis of down-regulated DEGs. **(B)** GO enrichment analysis of up-regulated DEGs. **(C)** KEGG enrichment analysis of down-regulated DEGs. **(D)** KEGG enrichment analysis of up-regulated DEGs.

Supplementary Figure 4 Establishment of necroptosis-related gene signature. **(A)** TCGA-SARC cohort was divided into two groups according to the median. **(B)** GSE17118 cohort was divided into two groups according to the median. **(C, E)** Principal component analysis (PCA) and t-distributed stochastic neighbor embedding (t-SNE) of TCGA-SARC cohort. **(D, F)** Principal component analysis (PCA) and t-distributed stochastic neighbor embedding (t-SNE) of GSE17118 cohort.

Supplementary Figure 5 Subgroup analysis of the risk scores in different clinical characteristics groups in TCGA-SARC cohort. **(A–K)** Subgroup analysis of risk score in different clinical characteristics groups including age, gender, site and histology.

Supplementary Figure 6 The development and validation of glycolysis-related gene signature. **(A)** OS curve of TCGA-SARC cohort based on glycolysis-related gene signature. **(B)** Disease-free survival (DFS) curve of GSE17118 cohort. **(C)** Evaluating the prognostic performance of the risk score using time-dependent ROC in TCGA cohort. **(D)** Evaluating the prognostic performance of the risk score using time-dependent ROC in GSE17118 cohort.

Supplementary Figure 7 **(A–E)** The coherence of key DEGs in the gene signature and immune infiltration.

Supplementary Figure 8 The application of risk score in other cancers. **(A–G)** The KM plot, distribution of risk score and clusters in liver cancer (LIHC), lung adenocarcinoma (LUAD), kidney clear cell carcinoma (KIRC), head and neck cancer (HNSC), colon cancer (COAD), endometrioid cancer (UCEC) and kidney papillary cell carcinoma (KIRP) in TCGA database.

## Abbreviations

STSs, soft tissue sarcomas; SARC, sarcoma; NRGs, necroptosis-related genes; TCGA, The Cancer Genome Atlas; GTEx, The Cancer Genome Atlas; GEO, Gene Expression Omnibus; DEGs, differentially expressed genes; AUC, area under the curve; ROC, receive operating characteristic; LASSO, the least absolute shrinkage and selection operator; KM, Kaplan-Meier; GO, Gene Ontology; KEGG, Kyoto Encyclopedia of Genes and Genomes; C1, cluster 1; C2, cluster 2.
