# Supplementary figures and images for: Comprehensive Profiling Reveals Prognostic and Immunogenic Characteristics of Necroptosis in Soft Tissue Sarcomas
Source: Front Immunol. 2022 May 18;13:877815. doi: 10.3389/fimmu.2022.877815 (PMC9159500; doi:10.3389/fimmu.2022.877815)

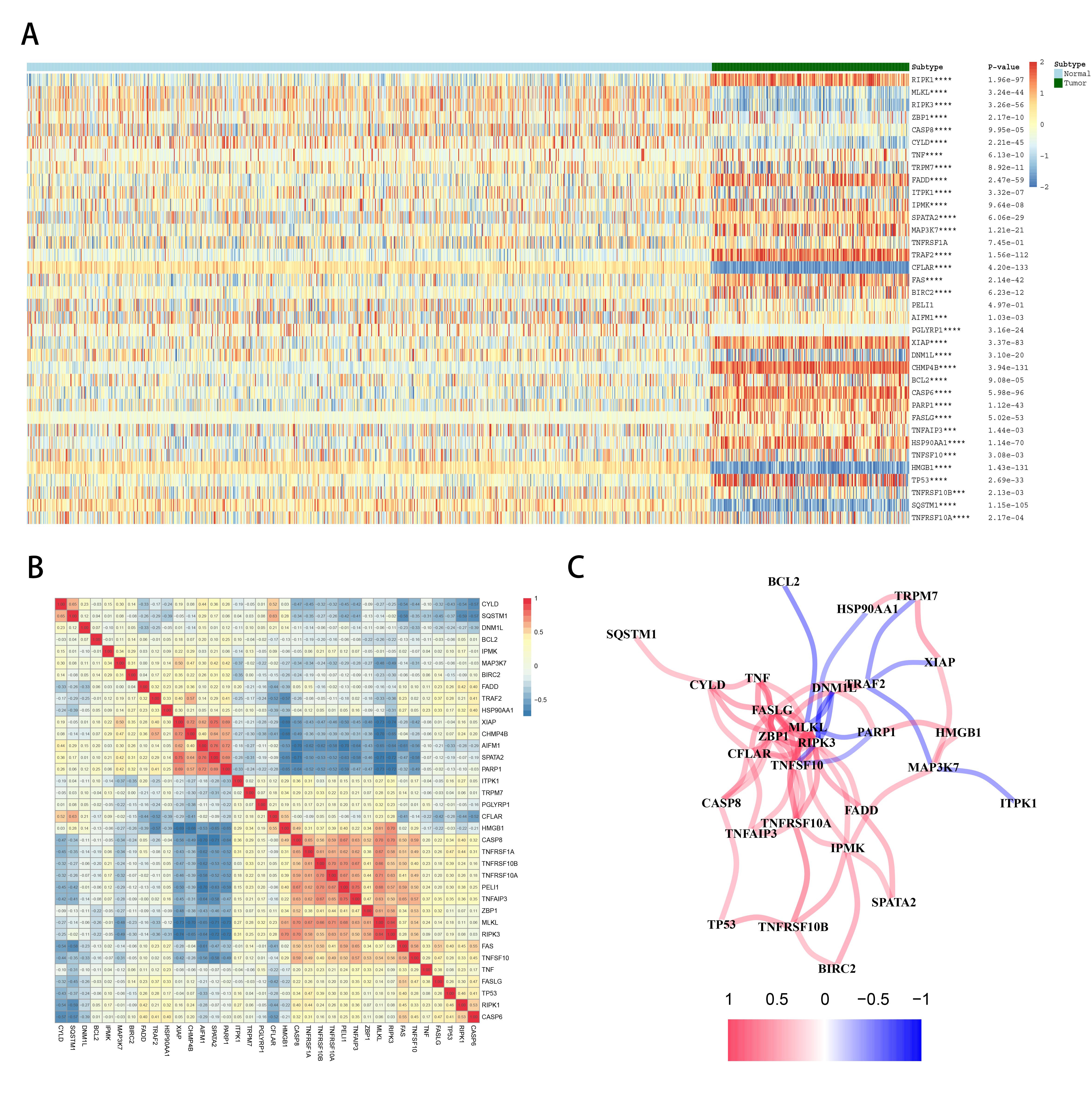

Supplement: Supplementary Figure 1 — Identification of NRGs between STSs and normal tissues. (A) NRGs-related heatmap of tumor tissue and normal tissue. (B, C) The correlation network of 34 NRGs. [file Image_1.jpeg]

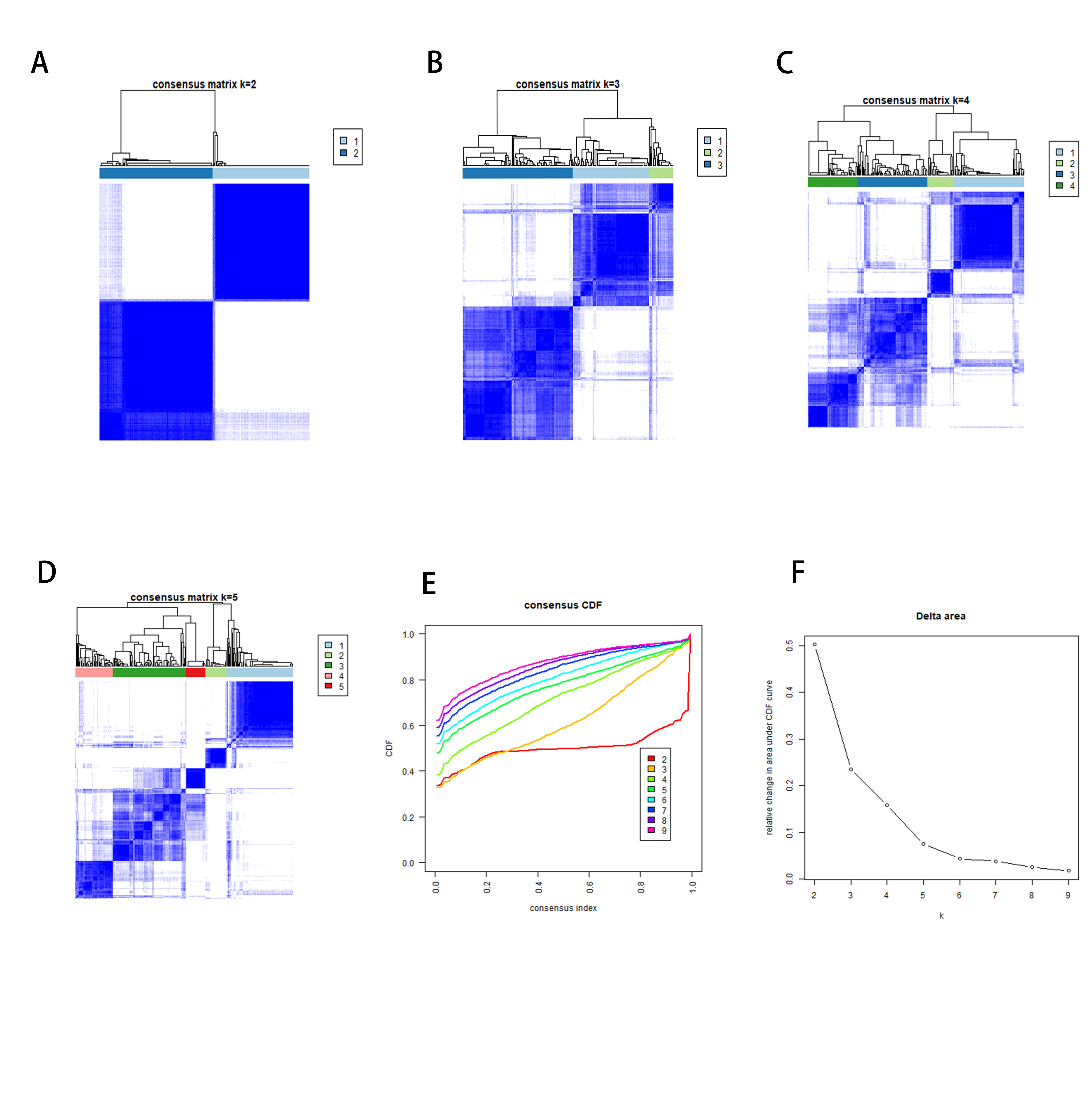

Supplement: Supplementary Figure 2 — Consensus clustering analysis. (A–D) Consensus clustering based on regulator-related DEGs (K = 2-5). (E) The CDF Plot based on the DEGs. (F) Delta area plot of consensus clustering based on the DEGs. [file Image_2.jpeg]

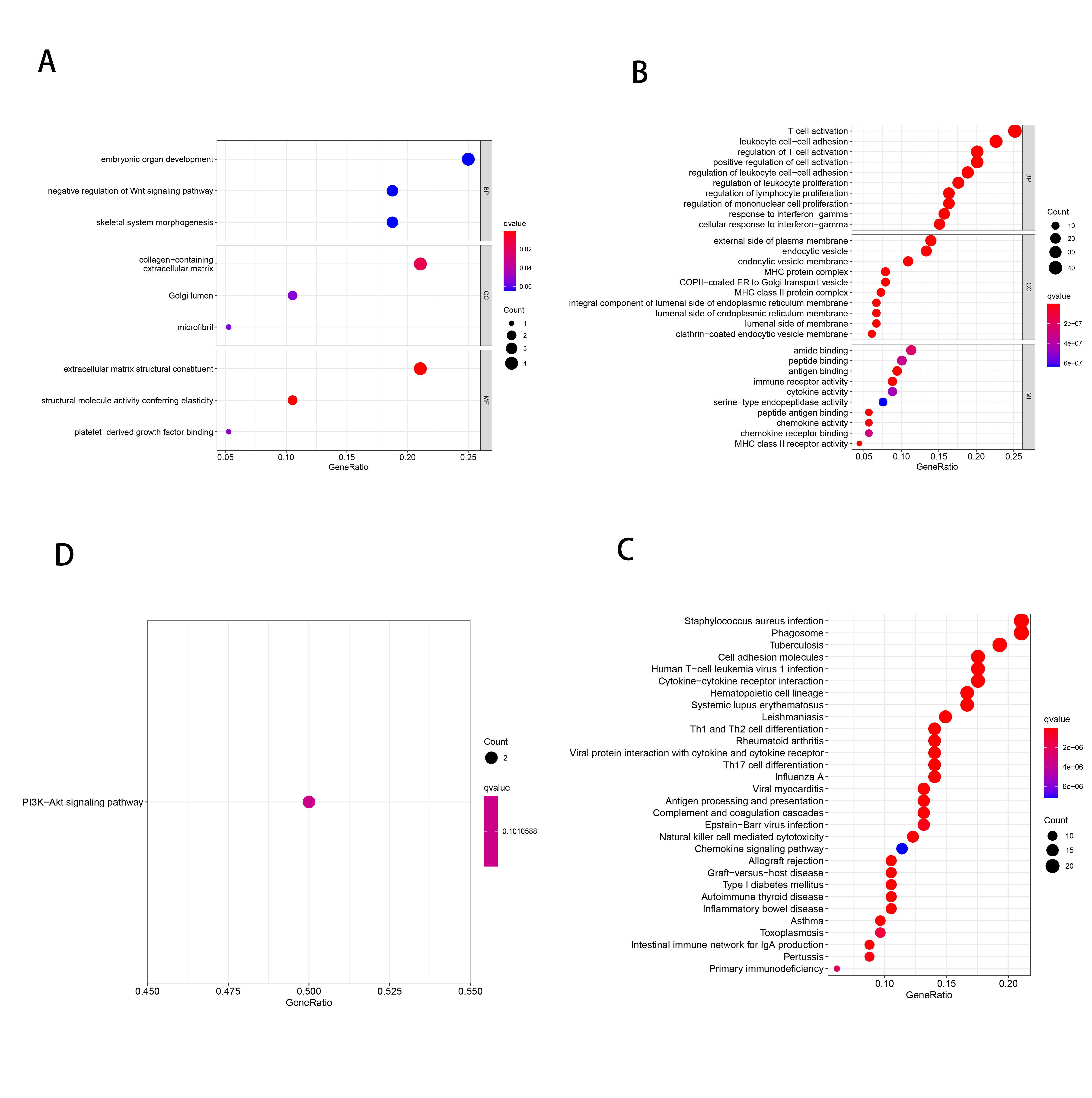

Supplement: Supplementary Figure 3 — Gene set enrichment analysis. (A) GO enrichment analysis of down-regulated DEGs. (B) GO enrichment analysis of up-regulated DEGs. (C) KEGG enrichment analysis of down-regulated DEGs. (D) KEGG enrichment analysis of up-regulated DEGs. [file Image_3.png]

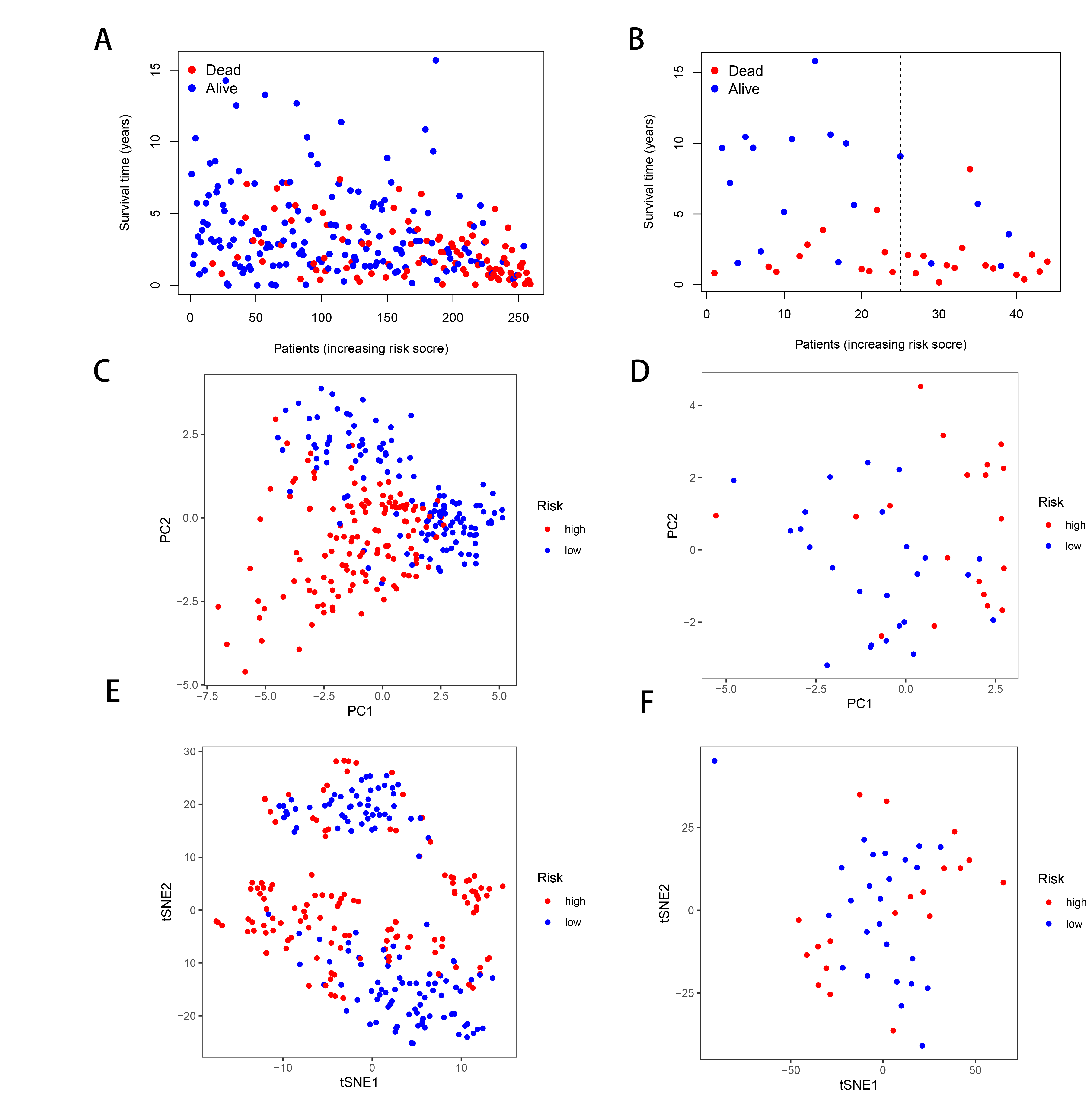

Supplement: Supplementary Figure 4 — Establishment of necroptosis-related gene signature. (A) TCGA-SARC cohort was divided into two groups according to the median. (B) GSE17118 cohort was divided into two groups according to the median. (C, E) Principal component analysis (PCA) and t-distributed stochastic neighbor embedding (t-SNE) of TCGA-SARC cohort. (D, F) Principal component analysis (PCA) and t-distributed stochastic neighbor embedding (t-SNE) of GSE17118 cohort. [file Image_4.jpeg]

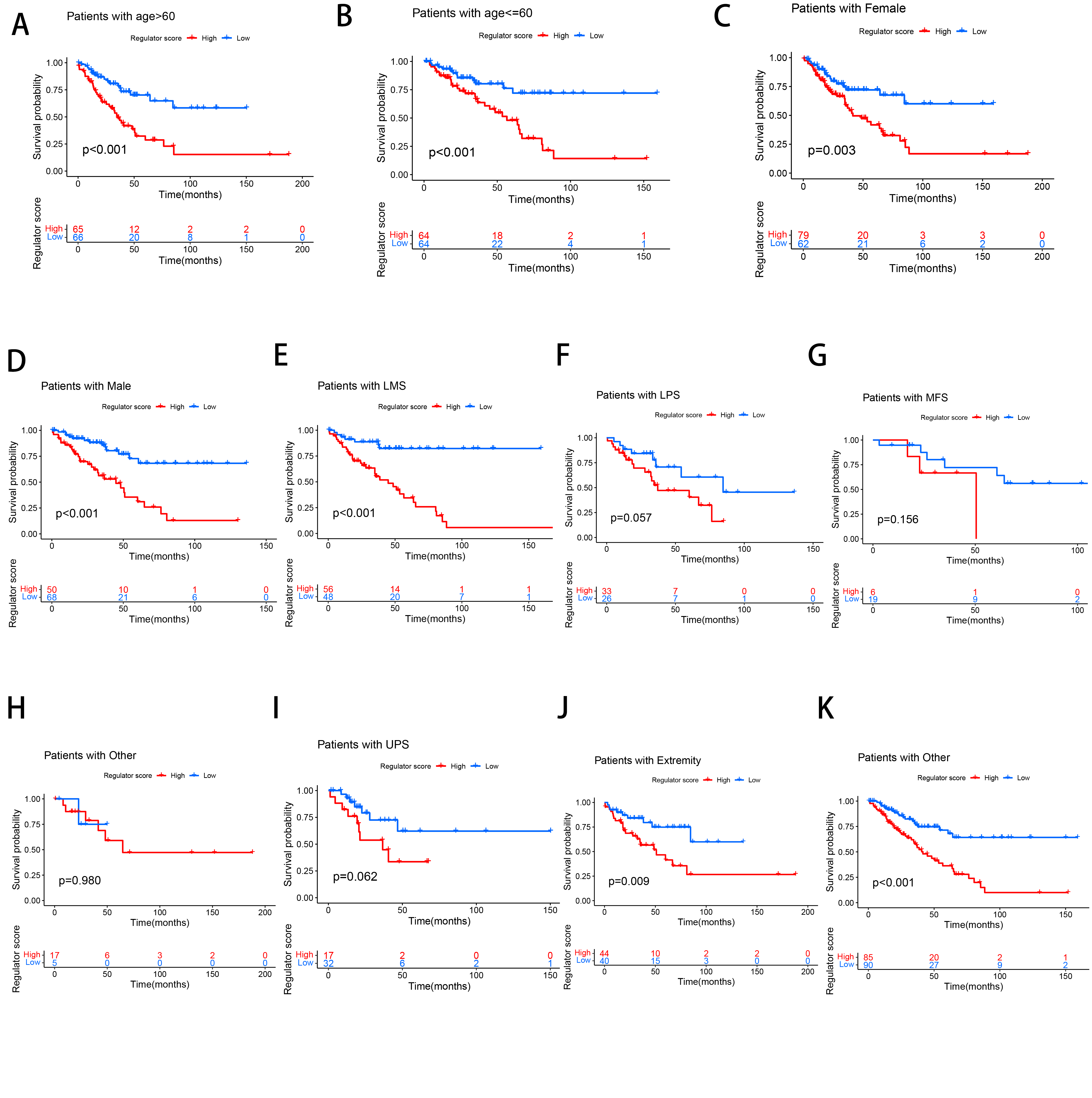

Supplement: Supplementary Figure 5 — Subgroup analysis of the risk scores in different clinical characteristics groups in TCGA-SARC cohort. (A–K) Subgroup analysis of risk score in different clinical characteristics groups including age, gender, site and histology. [file Image_5.jpeg]

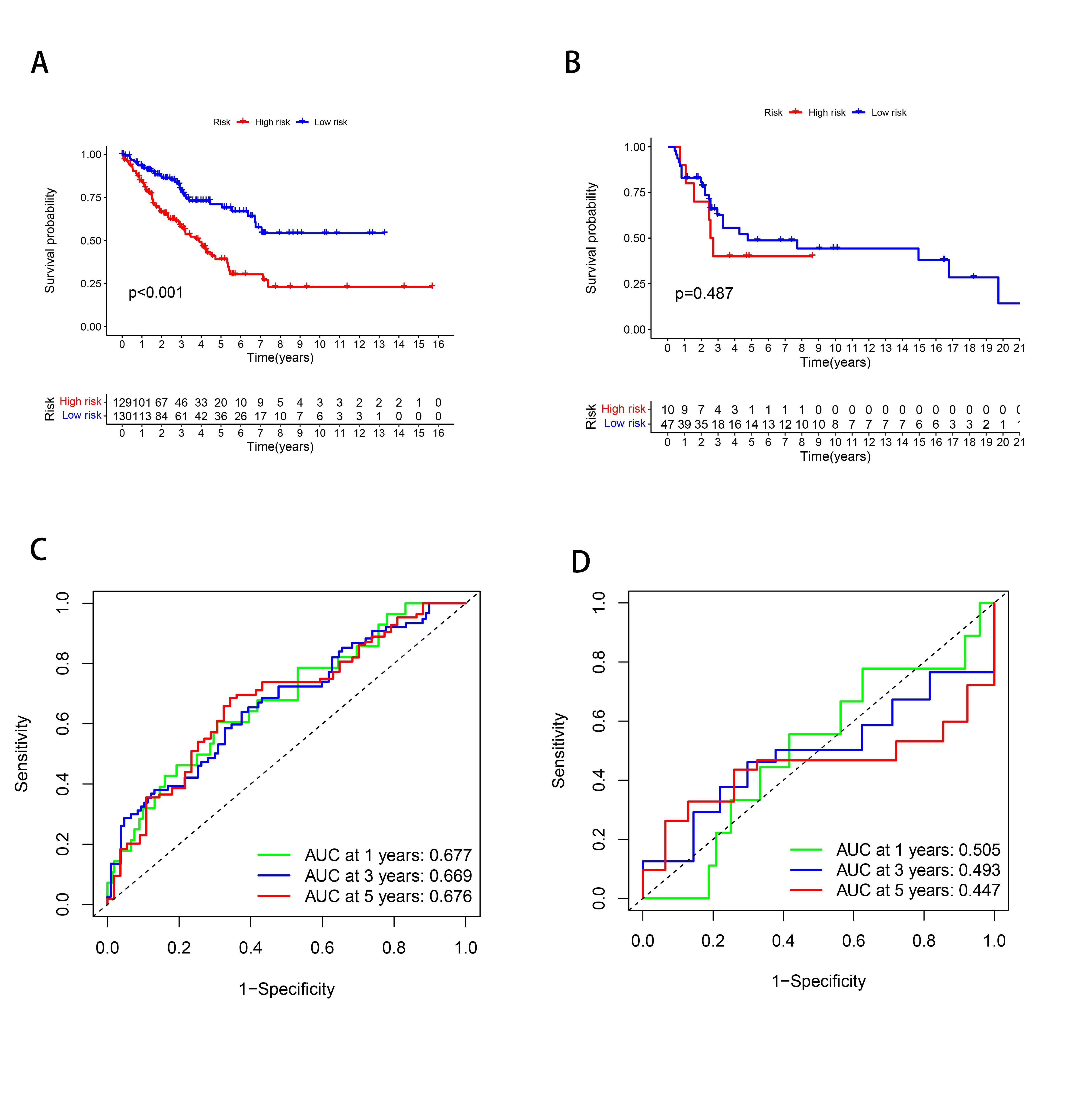

Supplement: Supplementary Figure 6 — The development and validation of glycolysis-related gene signature. (A) OS curve of TCGA-SARC cohort based on glycolysis-related gene signature. (B) Disease-free survival (DFS) curve of GSE17118 cohort. (C) Evaluating the prognostic performance of the risk score using time-dependent ROC in TCGA cohort. (D) Evaluating the prognostic performance of the risk score using time-dependent ROC in GSE17118 cohort. [file Image_6.jpeg]

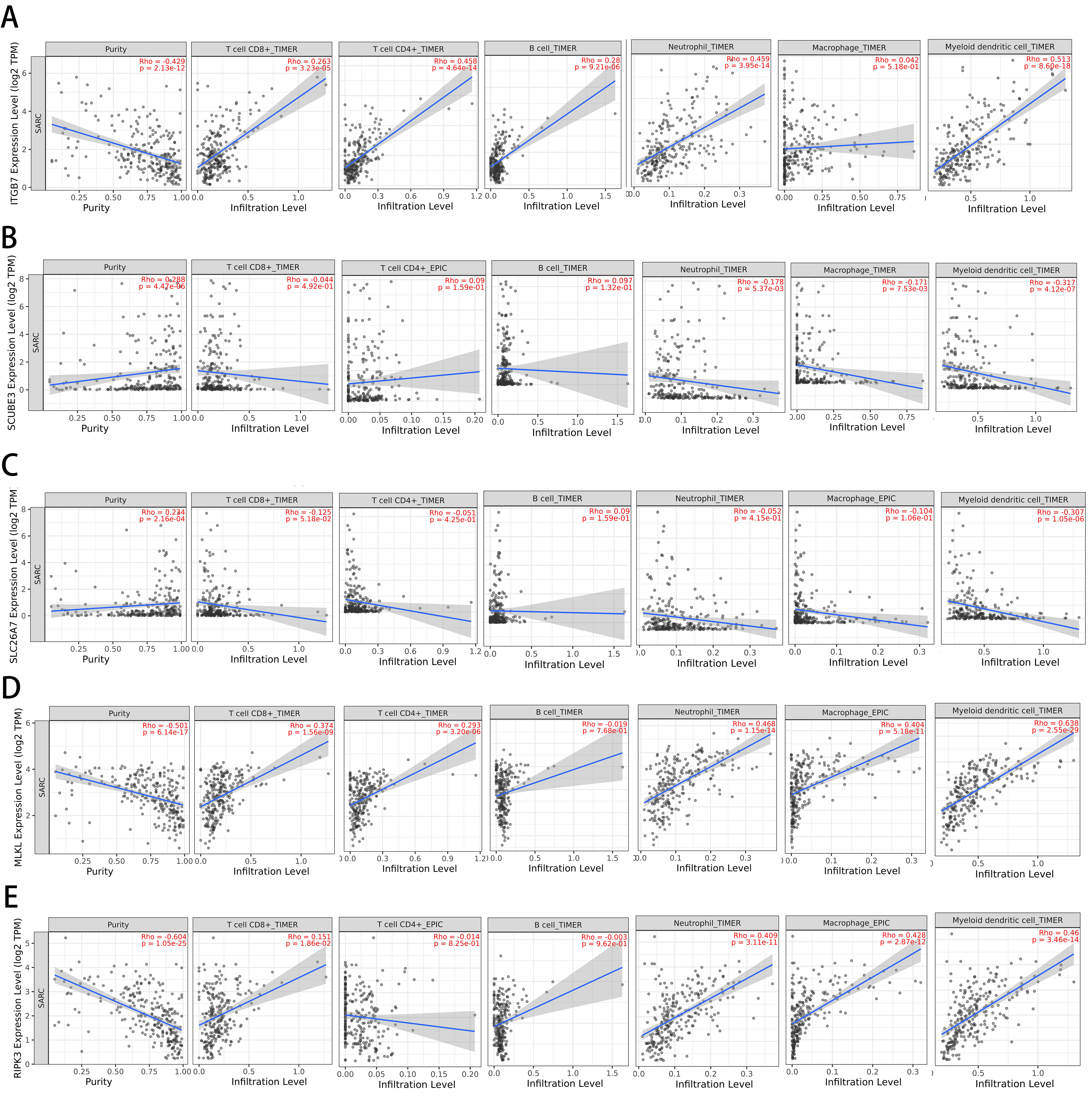

Supplement: Supplementary Figure 7 — (A–E) The coherence of key DEGs in the gene signature and immune infiltration. [file Image_7.jpeg]
